# Disparities in burden of disease in patients with rheumatoid arthritis across racial and ethnic groups

**DOI:** 10.1007/s10067-024-06869-9

**Published:** 2024-01-25

**Authors:** Jacqueline O’Brien, Sang Hee Park, Taylor Blachley, Maya Marchese, Nicole Middaugh, Keith Wittstock, Leslie R. Harrold

**Affiliations:** 1 CorEvitas, LLC, Waltham, MA USA; 2 Bristol Myers Squibb, Princeton, NJ USA; 3 University of Massachusetts Chan Medical School, Worcester, MA USA

**Keywords:** Disease burden, Health disparities, Longitudinal analysis, Race and ethnicity, Rheumatoid arthritis

## Abstract

To examine racial/ethnic differences in rheumatoid arthritis (RA) disease burden and change in clinical outcomes over time. We included CorEvitas Rheumatoid Arthritis Registry patients from two time periods (2013–2015 and 2018–2020). Clinical Disease Activity Index (CDAI) (as a continuous measure and as a dichotomous measure) and the Health Assessment Questionnaire-Disability Index (HAQ-DI) were assessed at each visit. Marginal means and their corresponding 95% confidence interval (CI) by race/ethnicity were estimated for each outcome using adjusted mixed effects linear and logistic regression models. Overall and pairwise tests were conducted to detect differences between race/ethnicity groups. Of 9,363 eligible patients (8,142 White, 527 Black, 545 Hispanic, 149 Asian), most (76%–85%) were female. At Visit 1, the mean disease duration ranged from 9.8–11.8 years. Estimated CDAI was significantly higher for Hispanics compared to Whites at Visit 1 (11.1 vs. 9.9; pairwise *P* = 0.033) and Visit 2 (9.2 vs. 8.0, pairwise *P* = 0.005). Disease activity improved over the 5-year study period among all race/ethnicity groups, though Hispanics improved less than Whites. Disease activity improved over the 5-year period across all racial/ethnicity groups, and disparities between racial/ethnicity groups in disease activity and functional status did persist over time, suggesting that further effort is needed to understand the drivers of these discrepancies to close this race/ethnicity gap.

**Table:** 

**Key Points** • *Disease activity improved over the 5-year period across all racial and ethnic groups.* • *Disparities between racial and ethnic groups in disease activity and functional status did persist over time, suggesting that further effort is needed to understand the drivers of these discrepancies and close this racial gap.*

**Supplementary Information:**

The online version contains supplementary material available at 10.1007/s10067-024-06869-9.

## Introduction and objective^1^

Rheumatoid arthritis (RA) is a chronic, inflammatory disease affecting long-term physical function and quality of life [1]. Between 1980–2019, the global prevalence of RA was 460 per 100,000 population [2]. Given more awareness and messaging of the treat-to-target (T2T) model, the last decade has witnessed improvements in RA treatment and management [3]. Despite these advances, a gap remains in the research regarding differences in clinical outcomes by race/ethnicity.

Recent studies indicate differential risk of RA by different demographic groups (e.g., race/ethnicity, socioeconomic status (SES), educational attainment) [4]; variances in use patterns of biologic medications for different racial/ethnic groups [5]; and lower functional status across SES quintiles within these groups [6]. Additional studies found disparities in time from symptom onset to first RA treatment, especially among Hispanics [7]. However, there is still a lack of large-scale studies investigating these disparities in clinical and patient-reported outcomes (PROs) of RA by race/ethnicity groups [8].

Previous research using the CorEvitas RA Registry found that although all groups improved over a five-year period (2005–2007 to 2010–2012), disparities in clinical outcomes and disease activity remained between White patients and patients from other race/ethnicity groups [9]. A need exists for a more current look at potential disparities in RA clinical outcomes, especially given increased awareness of T2T strategies. Thus, the objective of this study was to evaluate differences in disease burden and clinical outcomes for RA patients in the CorEvitas RA Registry from 2013–2020, by race/ethnicity group (White, Black, Hispanic, Asian). We address this question by looking cross-sectionally at data from two time points and by evaluating change over time in a longitudinal analysis.

## Method

### Data source

The CorEvitas RA Registry (formerly Corrona) is an ongoing, longitudinal clinical registry established in 2001 [10]. As of March 31, 2022, the Registry included 217 private and academic active clinical sites with 931 physicians throughout 42 states in the United States (US). Participating patients are required to have rheumatologist-diagnosed RA; be ≥ 18 years of age; and started or switched to an eligible systemic RA treatment at enrollment or within 12 months prior to enrollment. Data are collected using CorEvitas questionnaires from patients and providers during routine office visits approximately every six months.

### Study cohorts

Patients in the current study also must have had: at least one registry visit between January 1, 2013, and December 31, 2015 (the first visit in this time period is defined as Visit 1); at least one registry visit between January 1, 2018, and December 31, 2020 (the last visit in this time period is defined as Visit 2); non-missing Clinical Disease Activity Index (CDAI) measured at Visit 1 and Visit 2; and self-reported race/ethnicity at enrollment into the RA Registry.

### Measures and study outcomes

Data included sociodemographics, lifestyle characteristics, history of comorbidities, disease activity, treatment history (i.e., nonbiologic and biologic disease modifying antirheumatic drugs (DMARDs)), and PROs (i.e., Health Assessment Questionnaire-Disability Index (HAQ-DI) scores). The primary outcome was disease activity level as measured by CDAI [11]. We also assessed the proportions in low disease activity (LDA) (LDA = CDAI ≤ 10, which includes patients in remission) and in remission (CDAI ≤ 2.8) at Visit 1 and Visit 2. The HAQ-DI [12] measured self-reported patient functional status. In a longitudinal analysis, we assessed changes in CDAI and HAQ-DI over the follow-up period. We also evaluated achievement of CDAI LDA (and remission) among those not in LDA (or remission) at Visit 1.

### Primary predictor variable

Patients' self-reported race and ethnicity in the questionnaire were based on standard categories used by the National Institute of Health (NIH): White, African American, Asian, American Indian or Alaskan Native, Native Hawaiian, or Other Pacific Islander [13]. Self-reported patient ethnicity was defined as either Hispanic/Latino or non-Hispanic/non-Latino. We developed four groups for our study: White (non-Hispanic), Black (non-Hispanic), Hispanic, and Asian.

### Statistical analysis

Patient characteristics were summarized at Visit 1 by race/ethnicity as frequencies for categorical characteristics and means (SD) for continuous characteristics. Mixed-effects linear and logistic regression models for each outcome variable were developed with race/ethnicity group as the primary independent variable, site as the random effect, and the following covariates: a) Model 1: Practice setting (private vs. academic); b) Model 2: patient sociodemographics including age, gender, smoking history, college education status, and insurance type; c) Model 3: markers of RA severity: seropositivity (rheumatoid factor (RF) and/or anti-cyclic citrullinated peptide (anti-CCP)), duration, presence of x-ray erosions, number of prior DMARDs (conventional synthetic (cs)DMARDs, biologic (b)DMARDs, and targeted synthetic (ts)DMARDs), and disability status; d) Model 4: current RA medication use methotrexate (MTX), Non-MTX csDMARD, b/tsDMARD, and prednisone; and e) Model 5: history of comorbidities including cardiovascular disease, serious infections*,* cancer*,* hypertension*,* diabetes*,* anxiety/depression*,* fibromyalgia*,* chronic obstructive pulmonary disease (COPD)*,* asthma*, a*nd interstitial lung disease/pulmonary fibrosis (models not shown). The final model included all covariates significantly associated at α = 0.05 with the outcome in Models 1–5 (see Online Resource). We report adjusted marginal means by race/ethnicity for CDAI and proportions of patients in LDA, remission, and HAQ-DI at each visit. We tested overall differences across groups, and pairwise difference with respect to White-non-Hispanic race, for each time period separately. In addition, for the longitudinal analysis, we reported adjusted marginal mean change in CDAI and HAQ-DI from Visit 1 to Visit 2, and reported adjusted proportion of achievement of LDA or remission by Visit 2. Statistical analyses were conducted using R (version 3.6.2; R Foundation for Statistical Computing, Vienna, Austria).

### Ethics

The study was performed in accordance with the Declaration of Helsinki and the Guidelines for Good Pharmacoepidemiology Practice (GPP). All participating investigators were required to obtain full board approval for conducting noninterventional research involving human subjects with a limited dataset. Sponsor approval and continuing review was obtained through a central Institutional Review Board (IRB), the New England Independent Review Board (NEIRB; no. 120160610). For academic investigative sites without authorization to use the central IRB, full board approval was obtained from their respective governing IRBs, and documentation of approval was submitted to CorEvitas, LLC before participation and initiation of any study procedures. All patients in the registry were required to provide written informed consent and authorization before participating.

## Results

### Cohort

Baseline characteristics are summarized in Table 1. This study included 9,363 eligible patients (8,142 White, 527 Black, 545 Hispanic, and 149 Asian). Most patients (75.9%—85.2%) were female, and mean age ranged from 55.1 years for Hispanic patients to 60.7 years for White patients at Visit 1. The most common comorbidities were histories of hypertension and serious infections, and mean disease duration ranged from 9.8 years to 11.8 years.

**Table 1:** Demographic and clinical characteristics of study population by race/ethnicity at Visit 1

	Visit 1 (2013 – 2015)			
	White	Black	Hispanic	Asian
Total *N*	8,142	527	545	149
Patient Demographics				
Age, *n*, mean (SD)	8,142 60.7 (11.6)	527 59.5 (11.7)	545 55.1 (13.0)	149 57.2 (12.3)
Female	8,142 6,181 (75.9%)	527 449 (85.2%)	545 452 (82.9%)	149 127 (85.2%)
Former smoker	8,115 2,870 (35.4%)	526 138 (26.2%)	544 127 (23.3%)	147 23 (15.6%)
Current smoker	8,115 1,122 (13.8%)	526 80 (15.2%)	544 59 (10.8%)	147 9 (6.1%)
College education	8,094 4,926 (60.9%)	523 307 (58.7%)	532 277 (52.1%)	148 115 (77.7%)
Private insurance	8,142 6,034 (74.1%)	527 325 (61.7%)	545 398 (73%)	149 122 (81.9%)
Medicare insurance	8,142 3,061 (37.6%)	527 214 (40.6%)	545 150 (27.5%)	149 33 (22.1%)
Medicaid insurance	8,142 249 (3.1%)	527 69 (13.1%)	545 41 (7.5%)	149 < 5
No insurance	8,142 116 (1.4%)	527 9 (1.7%)	545 12 (2.2%)	149 < 5
Markers of RA Severity				
Seropositivity^**a**^	5,309 4,018 (75.7%)	310 262 (84.5%)	384 314 (81.8%)	100 85 (85%)
Duration of disease, *n*, Mean (years) (SD)	8,142 11.8 (9.8)	527 9.8 (8.7)	545 11.1 (9.6)	149 10.6 (8.4)
Presence of x-ray erosions	7,500 2,628 (35.0%)	474 164 (34.6%)	510 197 (38.6%)	136 50 (36.8%)
Number of prior DMARDs, *n*, mean (SD)	8,142 1.5 (1.8)	527 1.3 (1.6)	545 1.6 (1.9)	149 1.5 (1.7)
Disabled	7,996 881 (11%)	518 97 (18.7%)	531 85 (16%)	145 11 (7.6%)
Current RA Treatment				
MTX use	8,142 5,010 (61.5%)	527 360 (68.3%)	545 318 (58.3%)	149 91 (61.1%)
MTX dosage (mg/week), *n*, mean (SD)	4,880 16.2 (7.0)	349 15.8 (5.3)	307 16.6 (5.9)	90 15.3 (5.1)
Non-MTX csDMARD use	8,142 2,373 (29.1%)	527 151 (28.7%)	545 141 (25.9%)	149 36 (24.2%)
b/tsDMARD use	8,142 4,626 (56.8%)	527 280 (53.1%)	545 338 (62%)	149 89 (59.7%)
Prednisone use	8,142 1,778 (21.8%)	527 137 (26%)	545 120 (22%)	149 23 (15.4%)
Prednisone dose (mg/day), *n*, mean (SD)	1,715 6.4 (4.9)	133 7.1 (5.0)	115 6.3 (6.0)	23 7.2 (6.7)
History of Comorbidities				
CVD	8,142 909 (11.2%)	527 54 (10.2%)	545 42 (7.7%)	149 6 (4%)
Cancer	8,142 649 (8%)	527 34 (6.5%)	545 30 (5.5%)	149 9 (6%)
Hypertension	8,142 2,640 (32.4%)	527 217 (41.2%)	545 146 (26.8%)	149 32 (21.5%)
Diabetes	8,142 626 (7.7%)	527 77 (14.6%)	545 60 (11%)	149 10 (6.7%)
Anxiety/Depression	8,142 1,310 (16.1%)	527 86 (16.3%)	545 101 (18.5%)	149 16 (10.7%)
Serious infections	8,142 4,924 (60.5%)	527 256 (48.6%)	545 296 (54.3%)	149 75 (50.3%)
Fibromyalgia	8,142 357 (4.4%)	527 23 (4.4%)	545 20 (3.7%)	149 < 5
COPD	8,142 181 (2.2%)	527 < 5	545 < 5	149 < 5
ILD/Pulmonary Fibrosis	8,142 44 (0.5%)	527 < 5	545 < 5	149 < 5
Asthma	7,495 329 (4.4%)	490 22 (4.5%)	508 24 (4.7%)	140 7 (5%)

The first visit in this time period, January 1, 2013 through December 31, 2015, is defined as Visit 1

Data are *n* (%) unless otherwise specified; *b/tsDMARD* biologic/targeted synthetic DMARD, *csDMARD* conventional synthetic DMARD, *SD* standard deviation, *MTX* methotrexate, *CVD* cardiovascular disease, *COPD* chronic obstructive pulmonary disease, *ILD* interstitial lung disease

^a^Seropositivity defined as RF or anti-CCP positive

### Cross-sectional analyses

In our adjusted analyses, we estimated higher mean CDAI for Hispanic patients compared to White patients at Visit 1 (11.1 vs. 9.9; pairwise *P* = 0.033) and Visit 2 (9.2 vs. 8.0, pairwise *P* = 0.005). Proportions of LDA and remission were similar across groups at Visit 1 (overall LDA *P* = 0.364, overall remission *P* = 0.379, respectively). Yet, at Visit 2, Hispanic patients had lower proportions in LDA and remission compared to White patients (LDA: 72.4% vs. 78.8%, pairwise *P* = 0.007; remission: 25.1% vs. 31.2%, pairwise *P* = 0.028). Further, differences were observed in the HAQ-DI across race/ethnicity groups, with White patients having lower HAQ-DI scores (indicating better functional status) at Visit 1 (overall *P* = 0.009) and Visit 2 (overall *P* = 0.002) (Table 2).

**Table 2:** Adjusted marginal mean estimates (95% CI) for each outcome measure by race/ethnicity

	Visit 1 (2013–2015)				Visit 2 (2018–2020)			
	Mean	95% CI	Pairwise *P*^a^	Overall *P*^a^	Mean	95% CI	Pairwise *P*^a^	Overall *P*^a^
CDAI score								
White	9.9	(8.7–11.1)	Referent	0.058	8.0	(7.0–8.9)	Referent	0.027
Black	10.2	(8.6–11.8)	0.632		8.3	(7.0–9.6)	0.491	
Hispanic	11.1	(9.5–12.6)	0.033		9.2	(8.0–10.4)	0.005	
Asian	11.7	(9.5–14.0)	0.069		9.0	(7.2–10.8)	0.225	
Proportion of patients in LDA (%)								
White	68.8	(63.5–73.6)	Referent	0.364	78.8	(73.8–83.1)	Referent	0.048
Black	64.2	(56.0–71.7)	0.146		76.3	(68.6–82.5)	0.324	
Hispanic	67.1	(59.5–74.0)	0.546		72.4	(64.5–79.1)	0.007	
Asian	63.2	(50.3–74.5)	0.313		76.1	(63.7–85.3)	0.575	
Proportion of patients in remission (%)								
White	27.2	(22.9–31.8)	Referent	0.379	31.2	(26.8–36.0)	Referent	0.052
Black	24.8	(18.7–32.2)	0.440		26.5	(20.4–33.6)	0.114	
Hispanic	26.1	(20.0–33.3)	0.718		25.1	(19.5–31.8)	0.028	
Asian	20.0	(12.8–29.8)	0.110		26.1	(17.8–36.6)	0.281	
HAQ-DI score								
White	0.68	(0.65–0.71)	Referent	0.009	0.76	(0.73–0.80)	Referent	0.002
Black	0.75	(0.67–0.82)	0.063		0.84	(0.77–0.92)	0.027	
Hispanic	0.78	(0.71–0.85)	0.003		0.87	(0.80–0.94)	0.001	
Asian	0.71	(0.59–0.83)	0.596		0.78	(0.66–0.90)	0.833	

The first visit in this time period, January 1, 2013 through December 31, 2015, is defined as Visit 1 and the last visit during this time period, January 1, 2018 through December 31, 2020, is defined as Visit 2. ^a^Pairwise *P* is used to determine if there is a difference between means in patients in a specific race/ethnicity group compared with white patients; overall *P* is used to assess the differences in means across all race/ethnicity groups; See Online Resource for details on variables included in each final model. *CDAI* clinical disease activity index, *CI* confidence interval, *HAQ-DI* health assessment questionnaire—disability index, *LDA* low disease activity

### Longitudinal analyses

**Fig. 1:**
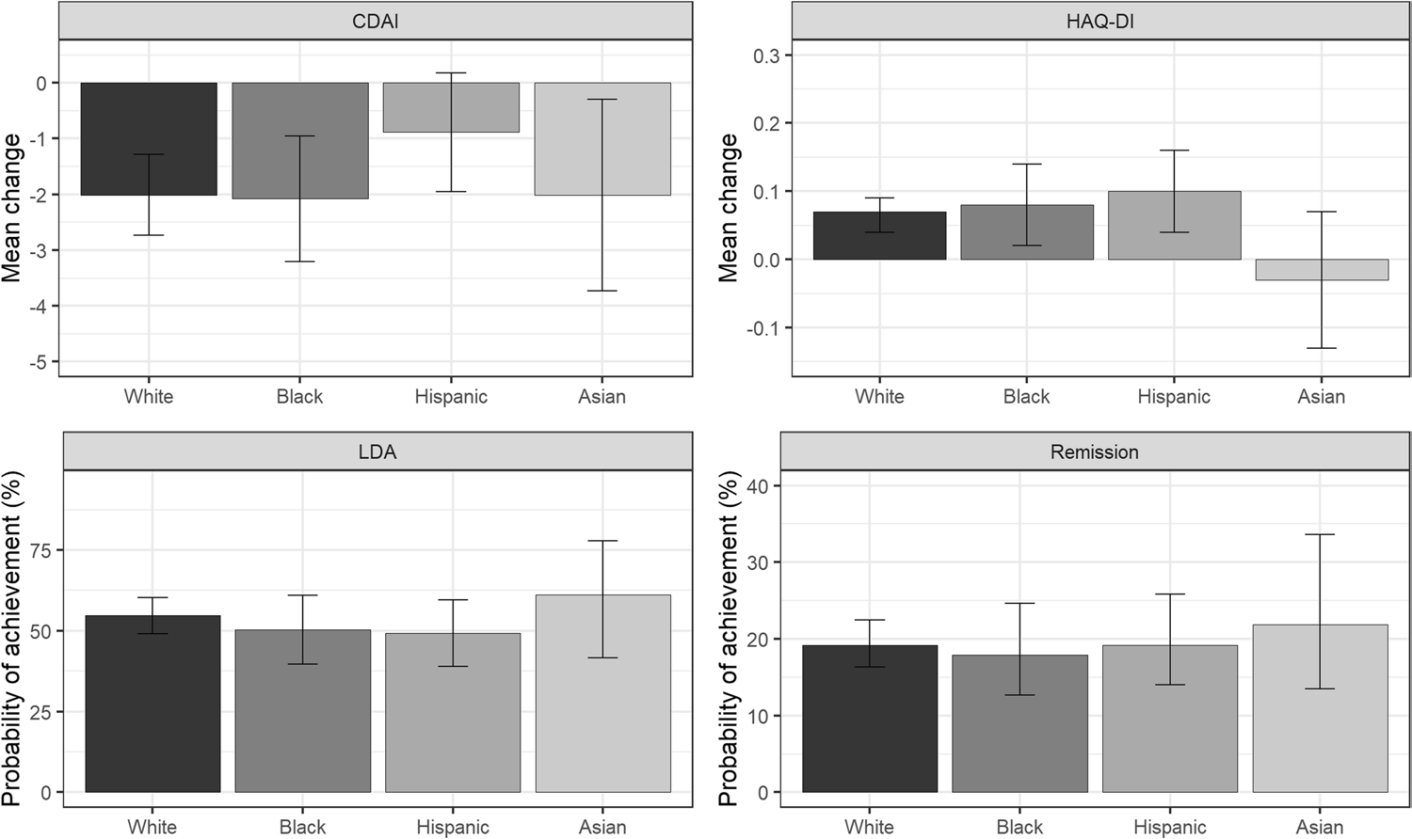
Adjusted marginal mean change in CDAI and HAQ-DI from Visit 1 to Visit 2, and adjusted probabilities of achievement of LDA and remission at Visit 2 by race/ethnicity. See Online Resource for details on variables included in each final model

In the final adjusted models, CDAI scores improved for all race/ethnicity groups from Visit 1 to Visit 2 (overall *P* = 0.077), though Hispanic patients improved significantly less than White patients (pairwise *P* = 0.010). There were no statistically significant differences in LDA or remission across groups (overall *P* > 0.1 for both) and no statistically significant differences in change in HAQ-DI over the follow-up period (overall *P* > 0.10) (Fig. 1).

## Conclusions

### Discussion

All race/ethnicity groups improved in disease activity over time in this study. Still, disparities in disease activity and functional status persisted. Hispanic patients had slightly higher CDAI than White patients at both time points. There were modest differences in self-reported physical function (HAQ-DI) including lower self-reported functional status for Hispanic patients compared to White patients (Visit 1) and Black and Hispanic patients compared to White patients (Visit 2). Further, magnitude of improvement was smaller for Hispanic patients than White patients.

Observed patterns were similar to the previous CorEvitas Registry study conducted in patients from 2005–2012 [9]. Both studies demonstrated evidence of more controlled disease (either by estimated mean CDAI, proportion in LDA/remission, or HAQ-DI) in White or Asian patients compared to Hispanic and Black patients. Additionally, both studies showed improvement over time across all race/ethnicity groups. Despite treatment advances and increased emphasis on treat-to-target approaches in more recent years, gaps between the groups persist.

This study could not address access to care concerning SES, as the study population had access to rheumatologists and specialty care. Other studies have looked at SES disparities in clinical outcomes. In a US national evaluation of functional status outcomes in patients with RA, the probability of functional decline over two years was significantly higher in patients with lower SES than in patients with high SES (18.9% vs. 14.1%), respectively [6]. Further, the probability of functional decline, as assessed by the Multidimensional Health Assessment Questionnaire, the HAQ-DI, and the Health Assessment Questionnaire-II, was higher in Hispanics (18.5, 95% CI 16.0–21.0) than in Whites (16.0, 95% CI 14.5–17.5). The relationship between SES and health remains complicated and future studies are needed.

Research has shown implicit racial/ethnic bias among healthcare professionals, with positive attitudes toward White patients and negative attitudes toward people of color [14]. Implicit bias may be associated with when minority patients first seek treatment. Riad et al. [7] found that the time to first treatment differs by race/ethnicity groups. Patients living longer with uncontrolled disease may experience substantial effects from long-term inflammation, including more extensive joint damage and increased disability. This circumstance may have occurred in this study cohort and may explain the higher mean CDAIs, or the smaller magnitude of CDAI improvement.

### Strengths and limitations

This study has numerous important strengths. The registry is a rich data source, following RA patients for many years and systematically collecting information about patient characteristics, clinical progress, and medications. The data collected on disease activity scores and PROs is not available in other non-randomized controlled trial (RCT) settings (e.g., claims databases), and since our study population includes patients from clinics, is more representative of the RA patient population than RCTs.

As in all observational studies, one limitation is the potential for residual confounding. However, this study considered many variables a priori and applied a stepwise approach to adjust for several demographic variables, as well as measures of comorbidities, disease activity, and treatment history. This study does lack some information, including medication adherence or patient beliefs and attitudes toward treatment, which could potentially differ between the groups. For instance, there could be differences in treatment uptake between the groups. The questionnaire did not collect information on certain SES variables, such as occupation, income, information on residence location, or proximity to care. Our study period did coincide with the Covid-19 pandemic; our requirement of a second visit between 2018–2020 could have selected subjects who were less impacted by the pandemic, which can vary based on race/ethnicity. Thus, our estimates by race/ethnicity could underestimate differences in the general population. Finally, the study population is highly educated, predominantly white, and had access to specialty care for RA, which limits generalizability to all RA patients.

Recruitment of diverse populations continues to challenge in RA research, which has focused predominantly on White populations. Researchers need more information on the epidemiology of RA, the disease course, and PROs in patients of different racial and ethnic backgrounds. Factors contributing to health inequity include access to care, SES, systemic racism, and other social determinants of health. Overall, patients, clinicians, researchers, health care organizations, and communities need a complex, multi-factorial solution to address health disparities in RA. This solution will include recruiting diverse populations into research, identifying access to care issues, and addressing systemic biases in our healthcare system.

### Summary

In conclusion, disease activity improved over time among all race/ethnicity groups, yet within a population of people who have access to a clinic and treatment, differences in disease activity and functional status by race/ethnicity remain. The differences may be minor, but even subtle differences in large patient populations can significantly impact disease burden. Further effort is needed to understand the drivers of these persistent discrepancies and close this gap.

### Supplementary Information

Below is the link to the electronic supplementary material.

Supplementary file1 (DOCX 27 KB)

## Data Availability

Data are available from CorEvitas, LLC, through a commercial subscription agreement and are not publicly available. No additional data are available from the authors.

## Footnotes

1 An earlier version of this study was presented as O’Brien J, Park SH, Blachley T, Marchese M, Middaugh N, Han X, Wittstock K, Harrold LR. Disparities in Burden of Disease in Patients with RA across Racial and Ethnic Groups. Poster presentation to ACR Convergence, Virtual, November 5–9, 2021.
