# Supplementary material for: Disparities in burden of disease in patients with rheumatoid arthritis across racial and ethnic groups
Source: Clin Rheumatol. 2024 Jan 25;43(3):921–7. doi: 10.1007/s10067-024-06869-9 (PMC10876763; doi:10.1007/s10067-024-06869-9)
Supplement: Supplementary file 1 — Supplementary file1 (DOCX 27 KB) [file 10067_2024_6869_MOESM1_ESM.docx]

Supplement

**Disparities in Burden of Disease in Patients with Rheumatoid Arthritis Across Racial and Ethnic Groups**

*Clinical Rheumatology*

Jacqueline O’Brien,^1^ Sang Hee Park,^2^ Taylor Blachley,^1^ Maya Marchese,^1^ Nicole Middaugh,^1^ Keith Wittstock,^2^ Leslie R. Harrold^1,3^

^1^CorEvitas, LLC, Waltham, MA, USA; ^2^Bristol Myers Squibb, Princeton, NJ, USA; ^3^University of Massachusetts Chan Medical School, Worcester, MA, USA

Corresponding author: Jacqueline O’Brien, jobrien@corevitas.com

**Disparities in Burden of Disease in Patients with RA Across Racial and Ethnic Groups**

**Variables Included in Final Models**

Disease Activity Measured by CDAI (cross-sectional analysis, Table 2):

Final model includes: age, female, current smoker, former smoker, academic affiliation, college education, private insurance, Medicaid insurance, Medicare insurance, no insurance, seropositivity, duration of disease, number of prior DMARDs, b/tsDMARD use, prednisone use, disabled status, history of serious infections, history of diabetes, history of anxiety/depression, history of fibromyalgia, history of CVD, history of asthma, site (random effect).

Proportion in Low Disease Activity Measured by CDAI (cross-sectional analysis, Table 2):

Final model includes: age, female, academic affiliation, college education, private insurance, Medicaid insurance, Medicare insurance, no insurance, duration of disease, b/tsDMARD use, prednisone use, disabled status, history of serious infections, history of diabetes, history of anxiety/depression, history of fibromyalgia, history of CVD, history of asthma, site (random effect)

Proportion in Remission Measured by CDAI (cross-sectional analysis, Table 2):

Final model includes: age, female, academic affiliation, college education, private insurance, Medicaid insurance, Medicare insurance, no insurance, seropositivity, non-MTX DMARD use, b/tsDMARD use, prednisone use, disabled status, history of serious infections, history of diabetes, history of anxiety/depression, history of fibromyalgia, history of CVD, history of asthma, site (random effect).

Patient Functional Status as Measured by HAQ-DI (cross-sectional analysis, Table 2):

Final model includes: Age, female, academic affiliation, college education, private insurance, Medicaid insurance, Medicare insurance, no insurance, seropositivity, duration of disease, number of prior DMARDs, b/tsDMARD use, prednisone use, disabled status, history of serious infections, history of diabetes, history of anxiety/depression, history of fibromyalgia, history of CVD, history of asthma, site (random effect)

Change in CDAI (longitudinal analysis, Figure 1):

Final model includes: Visit 1 CDAI, age at Visit 1, female, college education at Visit 1, private insurance at Visit 1, Medicaid insurance at Visit 1, Visit 1, Medicare insurance at Visit 1, no insurance at Visit 1, seropositivity at Visit 1, duration of disease, number of prior DMARDs at Visit 1, disabled status at Visit 1, methotrexate use at Visit 1, prednisone use at Visit 1, biologic/JAK use at Visit 1, history of serious infections at Visit 1, history of diabetes at Visit 1, history of anxiety/depression at Visit 1, history of fibromyalgia at Visit 1, history of CVD at Visit 1, site (random effect).

Change in HAQ-DI (longitudinal analysis, Figure 1):

Final model includes: Visit 1 CDAI, age at Visit 1, female, college education at Visit 1, private insurance at Visit 1, Medicaid insurance at Visit 1, Visit 1, Medicare insurance at Visit 1, no insurance at Visit 1, seropositivity at Visit 1, duration of disease, number of prior DMARDs at Visit 1, disabled status at Visit 1, prednisone use at Visit 1, biologic/JAK use at Visit 1, history of serious infections at Visit 1, history of diabetes at Visit 1, history of anxiety/depression at Visit 1, history of fibromyalgia at Visit 1, history of CVD at Visit 1, history of hypertension at Visit 1, site (random effect).

Probability of achievement of LDA over seven-year period (longitudinal analysis, Figure 1):

Final model includes: Visit 1 CDAI, age at Visit 1, female, seropositivity at Visit 1, number of prior DMARDs at Visit 1, disabled status at Visit 1, prednisone use at Visit 1, biologic/JAK use at Visit 1, history of serious infections at Visit 1, history of anxiety/depression at Visit 1, history of fibromyalgia at Visit 1, history of CVD at Visit 1, site (random effect).

Probability of achievement of remission over seven-year period by race/ethnicity (longitudinal analysis, Figure 1):

Final model includes: Visit 1 CDAI, age at Visit 1, female, college education at Visit 1, private insurance at Visit 1, Medicaid insurance at Visit 1, Visit 1, Medicare insurance at Visit 1, no insurance at Visit 1, seropositivity at Visit 1, duration of disease, number of prior DMARDs at Visit 1, disabled status at Visit 1, methotrexate use at Visit 1, non-MTX DMARD use at Visit 1, prednisone use at Visit 1, biologic/JAK use at Visit 1, history of serious infections at Visit 1, history of diabetes at Visit 1, history of hypertension at Visit 1, history of anxiety/depression at Visit 1, history of fibromyalgia at Visit 1, history of CVD at Visit 1, history or asthma at Visit 1, site (random effect).
